# Distribution of optometric practices relative to deprivation index in Scotland

**DOI:** 10.1093/pubmed/fdx074

**Published:** 2017-07-19

**Authors:** Robin Legge, Niall C Strang, Gunter Loffler

**Affiliations:** Department of Life Sciences, School of Health and Life Sciences, Glasgow Caledonian University, Cowcaddens Road, Glasgow, UK

**Keywords:** public health, socioeconomic factors, optometry, eye-care, deprivation

## Abstract

**Background:**

The UK National Health Service aims to provide universal availability of healthcare, and eye-care availability was a primary driver in the development of the Scottish General Ophthalmic Services (GOS) model. Accordingly, a relatively equal distribution of optometry practices across socio-economic areas is required. We examined practice distribution relative to deprivation.

**Methods:**

672 practices were sampled from nine Health Boards within Scotland. Practices were assigned a deprivation ranking by referencing their postcode with the Scottish Index of Multiple Deprivation (SIMD) tool (Scottish Executive National Statistics: General Report. 2016).

**Results:**

Averaged across Health Boards, the share of practices for the five deprivation quintiles was 25, 33, 18, 14 and 11% from most to least deprived area, respectively. Although there was some variation of relative practice distribution in individual Health Boards, 17 of the 45 regions (nine Health Boards, five quintiles) had a close balance between population and share of practices. There was no clear pattern of practice distribution as a function of deprivation rank. Analysis revealed good correlation between practice and population share for each Health Board, and for the combined data (*R*^2^ = 0.898, *P* < 0.01).

**Conclusion:**

Distribution of optometry practices is relatively balanced across socio-economic areas, suggesting that differences in eye-examination uptake across social strata are unrelated to service availability.

## Introduction

Suggested by Hart in 1971 and referred to as ‘conventional wisdom’^1^ in the 21st century, the inverse care law^2^ states that those who need healthcare the most are the least likely to receive it. In deciding where to practice, a general practitioner (GP) will take into account their expected income and the availability of local amenities (cultural and otherwise). Consequently, GPs in England gravitate toward areas with higher health needs, but also ‘lower deprivation levels, a more pleasant environment and higher levels of amenities’.^3^ Recent evidence indicates that a similar proclivity for practice in less deprived areas may exist amongst optometrists based in England.^4^ Based on this one might expect an under-provision of optometric practices in areas of higher deprivation in Scotland, particularly when one considers that the inverse care law is thought to apply most rigorously in situations exposed to market forces^2^ (in this case, spectacle sales). Alternatively, the higher eye-examination fee available to practitioners in Scotland (compared to England) may result in a more even distribution of optometry practices across socio-economic groups. The current study tested these two hypotheses by analysing the provision of optometric practices across different deprivation strata in Scotland.

In 2006, the Scottish Government introduced free National Health Service (NHS) eye examinations to the Scottish population. The motivation was to make more efficient use of resources by shifting aspects of eye-care from general practice and hospitals to community optometrists, whilst improving uptake for eye examinations.^5^ These changes have been successful in generating increased uptake in Scotland,^6^ however, concerns have been raised about levels of uptake within deprived socio-economic groups. Reports suggest that individuals attending for eye examinations increased disproportionately amongst higher income groups and the most educated.^6^

The General Ophthalmic Services (NHS-funded) eye-examination should present equitable benefit across socio-economic strata but inequality in uptake could result from a number of factors, including a distorted distribution of optometry practices across Scotland. As the business model of optometric practice often incorporates spectacle sales, it has been suggested that a high number of practices are situated within affluent areas with under-representation at the other end of the socio-economic spectrum.^7^ Although such a trend has been observed in Northern England,^4^ the higher examination fee available in Scotland should foster a more equitable distribution of optometry practices across socio-economic groups, as the business model is less reliant on private eye-care and spectacle sales.^8^

To determine if uneven uptake relates to a bias favouring practices in less deprived areas, we analysed the distribution of practices across deprivation levels in nine Scottish Health Boards.

## Methods

Addresses of all optometric practices were obtained from nine Health Boards in Scotland representing 91% of its overall population (collated January 2015). Scottish Government estimates^9^ of population and geographic extent (area in hectares) of each Health Board can be found in Table 1. Businesses that solely provide domiciliary services were excluded, as location is not representative of the geographical scope of their service provision.

**Table 1:** Scottish Government estimates of population and area in hectares of each of the 9 included Health Boards^9^

Health board	Population	Area (Hectares)
Ayrshire and Arran	371 110	336 721
Dumfries and Galloway	149 940	643 640
Fife	367 260	132 797
Forth Valley	300 410	265 174
Grampian	584 240	874 491
Greater Glasgow and Clyde	1 142 580	111 041
Lanarkshire	663 310	224 471
Lothian	858 090	172 937
Tayside	413 800	755 179

Practice postcodes were converted into deprivation scores. The SIMD^10^ is the Scottish Government’s tool used to identify areas subject to deprivation, enabling a deprivation score to be assigned to any postcode. Ranking is defined by employment, income, health, education, geographic-access to services, crime and housing. The lower the score, the more deprived the area. We used the tool to assign every practice to a quintile from 1 to 5, with Quintile ‘1’ representing the most deprived postcodes in Scotland; for reasons of clarity, Quintiles 1 and 2 are referred to as the ‘most deprived’ and ‘second most deprived’, whereas Quintiles 4 and 5 are referred to as the ‘second least deprived’ and ‘least deprived’ in this study. Unlike a pre-existing study of practice distribution in Tayside,^7^ the use of mean deprivation scores was avoided. The use of mean deprivation scores is problematic since they do not rank on a linear scale; a Data Zone with a score of 50, for example, is not twice as deprived as a zone with a score of 100.

The distribution of practices was analysed using the percentage of practices within a given quintile and Health Board relative to the total number of practices in that quintile at a macro level (i.e. encompassing the nine Health Boards).

To estimate the population within different socio-economic areas, we calculated the respective number of Data Zones. Data Zones (as defined by the Scottish Government) are population-based areas, each containing around 750 residents. In urban areas, they can contain only a handful of streets, whereas more rural Data Zones can describe areas many square miles in size. As with optometric practices, we expressed the distribution of the population as the percentage of Data Zones in a quintile of a given Health Board relative to the total number of Data Zones in that quintile. This allowed us to compare each Health Board’s ‘share’ of practices with that Health Board’s share of population within individual quintiles e.g. an analysis between Lothian’s share of practices which are based in the most deprived quintile with Lothian’s share of population (Data Zones) residing in the most deprived quintile. Data are presented subsequently as the ratio between the two shares: the percentage of practices divided by the percentage of Data Zones, for each deprivation score and Health Board. A quotient of one represents equality where the share of practices matches the population share. A value below one suggests a relative lack of practices, a value above 1 an oversupply of practices. This shows any inter-locality (in)equality with regards to ophthalmic service provision in Scotland.

The concept of a percentage share of geographical areas follows Government guidelines.^10^ These state that, in an area comprising 300 Data Zones, if 30 zones are defined as belonging to the most deprived quintile, 10% of that area can be considered to fall within this quintile. Scottish Government deprivation ranking does not take accessibility of optometric practices into account: this means that our ‘share of practices’ variable will not co-vary with ranking, bolstering the validity of our conclusions.

## Results

### Part 1: Distribution of optometric practices across Scotland

**Fig. 1:**
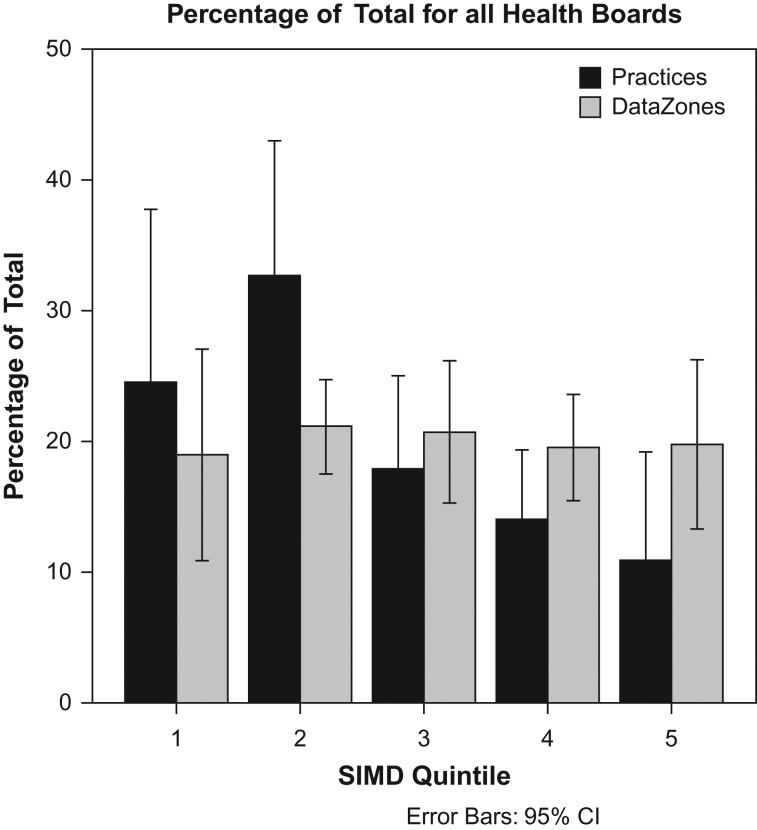
Percentage share of the number of practices and population (Data Zones) in each quintile relative to the total number of practices and population. The data are averaged across nine Health Boards. A percentage share of 1/5 = 20% indicates an equal distribution. Practices are relatively over-represented in the most deprived areas (lowest quintiles) and under-represented in the least deprived areas.

Figure 1 illustrates the percentage of practices and the percentage of population split into each of the five quintiles (1 = most deprived; 5 = least deprived). The data are averaged across the nine Health Boards. As expected, the proportion of population falling into each quintile is close to 20%. The percentage of practices shows an oversupply for the two most deprived quintiles (25 and 33%, respectively) and an under-supply for the two least deprived quintiles (14 and 11%, respectively). There are more practices available to the lower end of the socio-economic spectrum in Scotland than would be the case if practice provision was distributed exactly equally.

**Fig. 2:**
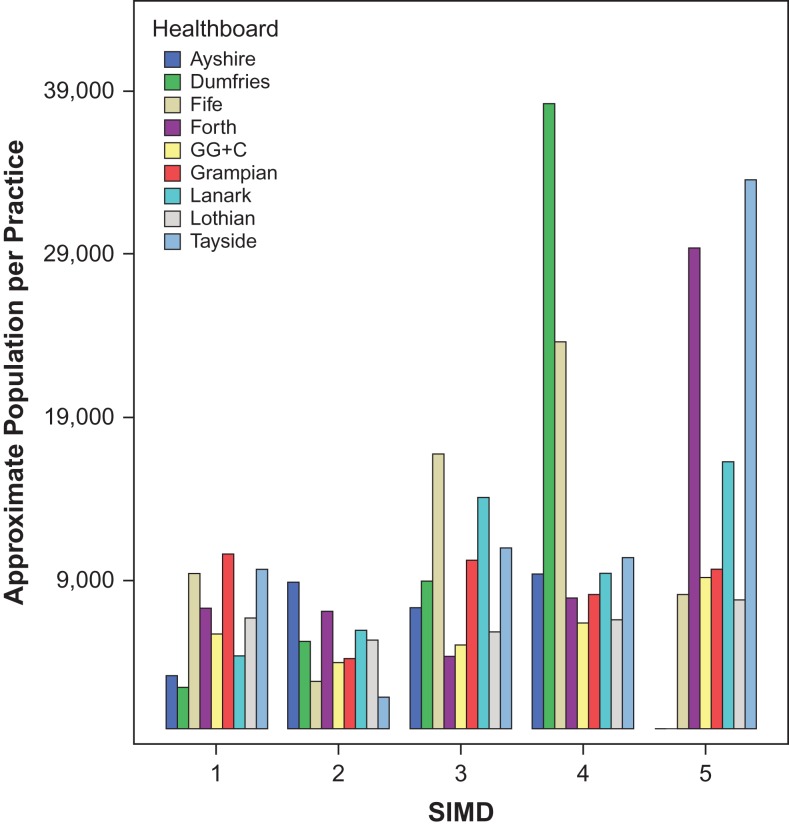
Approximate population per practice. Ayshire and Dumfries have no practices with Quintile 5 postcodes; as a result, there are seven bars for Quintile 5 in this figure (whilst the other four quintiles contain values for all nine included Health Boards). Health Boards are ordered alphabetically.

Figure 2 gives an indication of how many individuals are provided for by each practice within any given quintile within a Health Board. Population within the five quintiles of each health board were approximated by multiplying the number of Data Zones by the average population per zone (750 residents). This figure is then divided by the number of practices found in the same quintile of said Health Board to provide a guide to the number of people each practice is providing for.

In Fig. 2, the median number of patients supported by a practice in the nine Health Boards is 8063 (mean ± SD = 9618 ± 7333). As shown by the obvious outliers in the 2 least deprived quintiles (Dumfries in quintile 4; Forth Valley and Tayside in quintile 5), rural areas are more likely to be home to optometry practices which serve greater patient volumes (since there are very few practices in these areas).

**Fig. 3:**
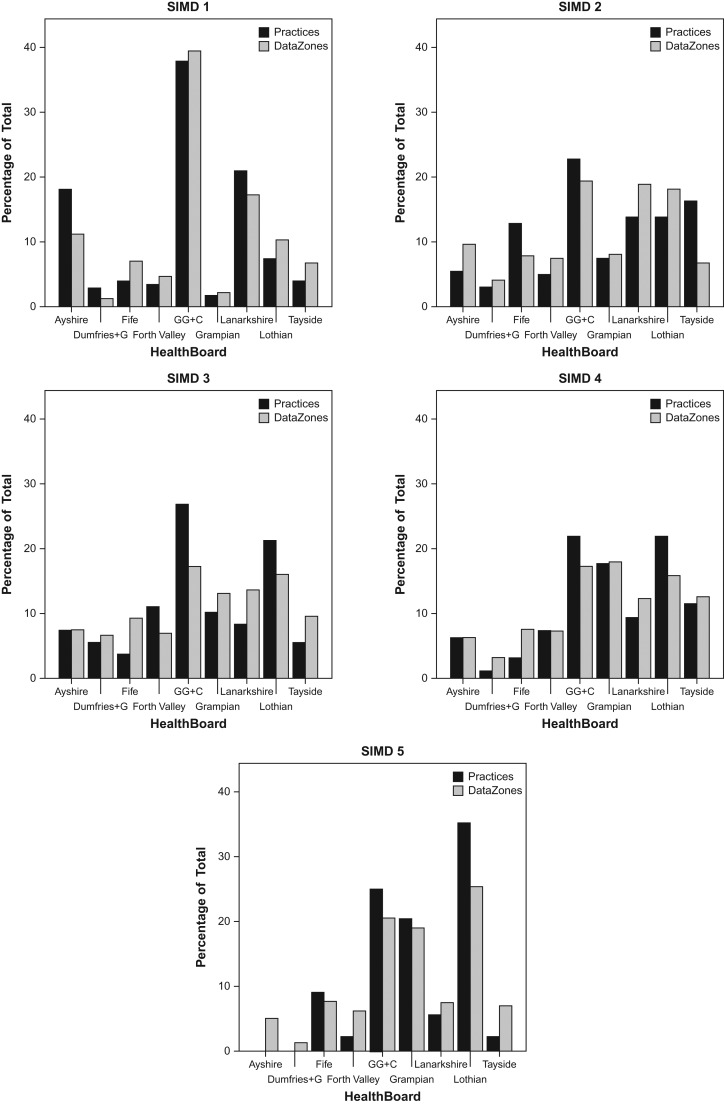
Percentage of all optometric practices in each quintile versus percentage of all Data Zones in each quintile, arranged by Health Board. In each chart grey bars represent % of total number of Data Zones whilst black bars represent % of total number of practices. This indicates, for example, that in instances where the black bar is smaller than the grey bar there may be an under-provision of practices within the corresponding Health Board.

Figure 3 illustrates the percentage share of each quintile’s practices and Data Zones at a national level contained within individual Health Boards. These figures enable one to assess inequalities between Scottish Health Boards in terms of provision.

Figure 3 is subdivided into five levels of deprivation (‘SIMD 1–5’). Within each panel, bars show the percentage of practices and Data Zones for each health board. The percentage data are relative to the total number of practices or Data Zones in that quintile across all Health Boards. For example, Greater Glasgow and Clyde, contains just under 40% of all practices and Data Zones in the most deprived quintile (Quintile 1) across Scotland. Presenting data in this way allows a direct comparison of (in-) equalities of eye-care provision across health boards separated by deprivation. For example, Fife shows an under-provision of optometric practices (black bar smaller than grey bar) in the most deprived quintile whereas Ayrshire shows an over-provision.

A correlation analysis assessed the relationship between practice share and population share in each quintile (all Health Boards combined). This examines if, for example, a higher share of Data Zones (population) within a quintile corresponds with a higher share of practices in that quintile. Table 2 shows a significant relationship between practice and population share (*P* < 0.05; *R*^2^ between 0.791 and 0.981). For each quintile, the percentage share of practices is highly correlated with percentage share of Data Zones. The relationship between practice share and Data Zone share holds across the five quintiles (*R*^2^ = 0.898, *P* < 0.001). This shows that for each quintile, areas with a higher population enjoy a higher share of practices.

**Table 2:** Pearson correlation (two-tailed) for each quintile, representing correlation between percentage of Data Zones in each quintile versus percentage of practices (for all health boards)

SIMD quintile	Pearson correlation (% of Data Zones versus % of practices)	Significance (*P*-value)
ALL	0.898	<0.001*
1	0.960	<0.001*
2	0.680	<0.05*
3	0.791	<0.05*
4	0.935	<0.001*
5	0.981	<0.001*

*Denotes significance at 0.05 level.

### Part 2: Distribution of optometric practices within health boards

Table 3 shows data for deprivation quintiles within Health Boards, presenting the ratio between the percentage share of practices and the percentage share of population in that quintile relative to the entire Health Board. It should be noted that the data used in Table 3 are not the same as those in Fig. 3. Figure 3 illustrates the percentage share of each quintile’s practices and Data Zones at a macro level (across all nine Health Boards). For example, of all the practices/zones in Scotland which fall within the most deprived quintile, the data show the percentage that are found in e.g. Ayrshire (18 and 11%, respectively). Here, Table 3 illustrates the share within Health Boards. For example, of all the practices/data zones in Ayrshire, the table considers the percentage found in the most deprived quintile. These percentages are then used to derive the practice/data zone ratio shown in the table. In contrast to the results presented in Part 1, data in the analysis here will not be affected by national trends in the distribution of practices or population across quintiles.

**Table 3:** Share of practices in each quintile versus percentage share of Data Zones within each quintile (columns) for different Health Boards (rows)

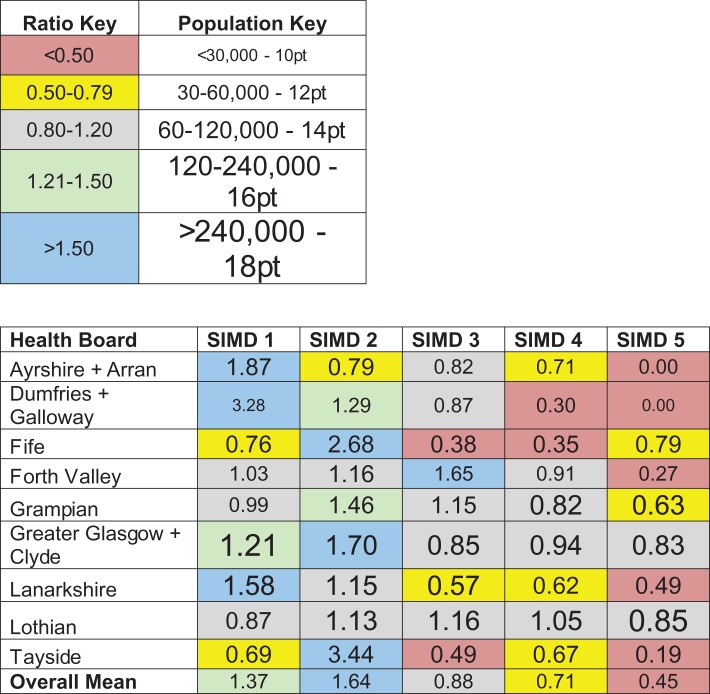

The data are expressed as a ratio quotient (%practices / %Data Zones). Red cells represent areas of low practice density. Blue cells indicate areas of high practice density. Colour-coding is in online version of manuscript only. Larger font sizes denote areas with higher populations.

A quotient of ‘1’ represents a 1 to 1 correspondence between the two factors; i.e. a balanced distribution of practices. Values greater than ‘1’ indicate that there are more practices than Data Zones (population) in terms of percentage share, with values below ‘1’ indicating the opposite. In Tayside, for example, there is an ~30% under-representation of optometric practices within the most deprived quintile (quotient 0.69) but a preponderance of practices within the second most deprived quintile (quotient 3.44, i.e. ~3.4 times more practices than equality). Individual cells were coloured to reflect this: areas with an essentially balanced share of practices and population (0.8 ≤ ratio ≤ 1.2) are presented on a grey background. Those with a lower practice than population share are set on a hot-coloured background (red < 0.50, yellow 0.50–0.79), those with an under-representation of practices on a cold background (green 1.21–1.50, blue > 1.50). The font size of the values in each cell reflects population size within that area, with larger font sizes denoting Health Boards with larger populations (for example, Greater Glasgow and Clyde is home to >240 000 people living in the most deprived quintile and this is indicated by using the largest font size in the pertinent cell of Table 3).

It is evident from the Table that a large number (*N* = 17) of areas fall close to the value that would be expected from a balanced practice distribution (between 0.8 and 1.2). However many areas are either substantially under- or over-represented. Values range from 3.44 in the most deprived quintile in Dumfries and Galloway (around 3.4 times more practice share than population share) to 0 (no practices) in the least deprived areas of Ayrshire & Arran and Dumfries & Galloway.

Although not all Health Boards follow the same pattern, the most frequent trend is over-representation of practices in the two most deprived quintiles and under-representation in the least deprived quintiles. Seven out of nine Health Boards have the highest concentration of practices in one of the two most deprived quintiles (exceptions being Forth Valley and Lothian) and all nine have the lowest concentration in one of the two least deprived quintiles. Average ratios (across Health Boards, bottom row in Table 3) reflect this pattern, with ~50% (37–64%) more practice share than population share in the two most deprived quintiles and ~42% (29–55%) less practice share than population share in the two least deprived quintiles.

## Discussion

### Main findings of this study

While there is inevitable variation across Health Boards in Scotland, our results show a largely equitable distribution of optometry practices across strata of deprivation (Fig. 1) with the share of practices in each quintile correlating highly with share of Data Zones when analysed at a national level (Table 2). This evidence suggests that the eye-care funding model in Scotland enables optometry practices to function in all socio-economic areas. These findings contrast with reports that optometry practices in Leeds, England are concentrated within the least deprived areas.^8^

At a local level, individual Health Board results (Table 3) show that practice distribution is not entirely even but is not linked to socio-economic scale: for example, within the most deprived quintile, Fife, Grampian, Lothian and Tayside have a density of practice distribution that falls below their respective population shares. In contrast, Ayrshire and Arran, Dumfries and Galloway, Forth Valley, Greater Glasgow and Clyde, and Lanarkshire all have an over-representation of practices in the most deprived quintile. An under-representation in this quintile does not appear to be indicative of a general trend in more deprived areas: all four Health Boards which have an under-representation in the most deprived quintile have an over-representation in the second most deprived quintile. The largest urban area in Scotland (Greater Glasgow and Clyde) has the highest ratios (greatest numbers of practices relative to population) in these two quintiles. This may result from a disproportionate number of practices in city-centre locations coupled with low socio-economic ranking of associated postcodes. Importantly, Table 3 indicates that optometric practice distribution is not skewed away from the most deprived quintiles. The average ratio for the two most deprived quintiles for each Health board is above 1 (range 1.0–2.29; mean ± SD = 1.50 ± 0.41). For the two least deprived quintiles, only one cell contains a ratio ≥1.

Comparison between our results for Tayside and an earlier study^7^ agree that a low practice density is found in the most deprived quintile. However, this information does not provide the full picture of optometry practice distribution in Tayside, or in Scotland in general. Table 3 shows that the greatest optometry practice density is in the second most deprived quintile, whilst the two least deprived quintiles possess a lower practice density than the most deprived. This suggests that practice distribution in Tayside is not concentrated in the least deprived areas.

### What is already known on this topic

A recent study examining the location of optometric practices in the Tayside area found an inequality in terms of optometric provision, concluding that the most deprived areas are home to the lowest numbers of practices.^7^ Similar results have been reported in the large metropolitan area of Leeds, England. People aged over 60 or under 16 from the least deprived quintile were more likely to attend for an eye-examination than persons in the same groups from the most deprived quintile (71 and 23%, respectively).^8^ As eye examinations are not generally free in England (unless the patient is over 60 or under 16) these age groups are apt for comparison to the Scottish data. Geographical distance to an optometric practice was suggested as one of the reasons for the lack of uptake by the most deprived quintile and previous work in Leeds has shown a mismatch between the most deprived areas and the locations of optometry practices.^4^ Since one would expect lower rates of rent in deprived areas to present some form of commercial incentive to any prospective practice-owner, evidence such as this suggests alternate drivers incentivising this lean toward a preponderance of optometric provision in less deprived areas.

### What this study adds

Prior to this study, the only evidence describing the distribution of optometric practices in Scotland relative to deprivation was limited to a small-scale study^7^ of a single quintile within a single Health Board (Tayside). Since the exclusive scope of the Tayside study presented a snapshot of information, which is not representative of wider practice distribution trends, it is important to be aware of the bigger picture in Scotland. Although our data agree that there is a low practice density in the most deprived quintile in Tayside, our broader findings suggest that any inequality in eye-care uptake between socio-economic groups in Scotland is unlikely to result from availability of services.

The finding that population share and optometric practice share correlate across quintiles suggests that NHS funding for sight tests in Scotland may be helping to facilitate the on-going commercial viability of practices in more deprived areas, despite the likely shortfall in sales of more profitable optical appliances. Lower rates for commercial property rental in deprived areas may also help such facilitation, although previous evidence from England^4^ (where the NHS only funds sight tests for those under 16 or over 60 years of age) suggests that this factor alone is not a sufficiently compelling driver to incentivise an equitable distribution of practices.

Contrary to some earlier reports on imbalances affecting individual parts of Scotland, a wider view shows a largely balanced provision of optometric practices across different socio-economic groups. Any difference in the uptake of eye examinations across social strata can, therefore, not be explained on the basis of optometric practice availability.

### Limitations of this study

The distribution of optometry practices is only one metric to inform on uptake and our conclusions are based on the assumption that individuals accessing services reside in the Data Zone where they attend for an eye-examination. However, there is evidence to suggest that patients are more likely to attend a practice in proximity to their home. Previous work in Tower Hamlets (London) found that eye-examination attendance drops sharply with a domicile-to-practice distance as short as 0.8 km.^11^ It should, however, be noted that this example may not be representative of trends in less deprived and rural areas. Undoubtedly some patients who live outside the city-centre will access eye-care services there, either due to proximity to their place of work or proximity to retail outlets. In Greater Glasgow and Clyde, city-centre postcodes (e.g. G1 or G2 postcodes) are not all defined by the same level of deprivation, with postcodes commonly belonging to Quintiles 2, 3 or 4. Such examples of city-centre variance in deprivation levels complicate the interpretation of the data presented here.

Two other reasons for a shortfall in uptake in eye-care between socio-economic groups have been proposed: the first is a social bias whereby individuals from more deprived social strata have a lower propensity to attend for eye examinations, perhaps explained by the cost of optical appliances,^12^ and a perceived pressure to buy glasses.^13^ The second is a lack of awareness regarding the availability of free eye examinations in some groups.^12^ Both reasons suggest that any inequality between socio-economic groups may be related to the utilization rather than provision of the eye-care system. Therefore it is important that the eye-care sector work together to improve awareness of the system and encourage uptake in all socio-economic groups. From a Scottish perspective this goal does not appear to require an establishment or re-distribution of optometric practices.
